# Atmospheric Pressure Plasma Surface Treatment of Polymers and Influence on Cell Cultivation

**DOI:** 10.3390/molecules26061665

**Published:** 2021-03-17

**Authors:** Hilal Turkoglu Sasmazel, Marwa Alazzawi, Nabeel Kadim Abid Alsahib

**Affiliations:** 1 Department of Metallurgical and Materials Engineering, Atilim University, Incek, Golbasi, 06830 Ankara, Turkey; 2 Department of Biomedical Engineering, Al Nahrain University, Al Jadriya Bridge, Baghdad 64074, Iraq; marwa.azzawi86@gmail.com (M.A.); n_K_alsahib@yahoo.com (N.K.A.A.)

**Keywords:** plasma, atmospheric pressure, cell cultivation, polymer, surface modification

## Abstract

Atmospheric plasma treatment is an effective and economical surface treatment technique. The main advantage of this technique is that the bulk properties of the material remain unchanged while the surface properties and biocompatibility are enhanced. Polymers are used in many biomedical applications; such as implants, because of their variable bulk properties. On the other hand, their surface properties are inadequate which demands certain surface treatments including atmospheric pressure plasma treatment. In biomedical applications, surface treatment is important to promote good cell adhesion, proliferation, and growth. This article aim is to give an overview of different atmospheric pressure plasma treatments of polymer surface, and their influence on cell-material interaction with different cell lines.

## 1. Introduction

In the past decades, scientific advances in tissue engineering have created a unique opportunity to fabricate or improve biological substances that replace, repair, maintain, or enhance tissue functions. Biomaterial engineering is an essential aspect of the tissue engineering field that relies on a combination of materials, cells, and bioactive molecules; to cause a desirable cell-biomaterial interaction within the host environment [1,2]. Polymers have been widely utilized in biomedical applications due to their bulk properties including physical, chemical, and biological properties. In addition to bulk properties, specific surface properties, such as surface free energy, hydrophilicity, and surface morphology, are required in particular medical applications [3,4]. However, polymers do not possess the desirable surface properties required for these applications [5].

**Figure 1:**
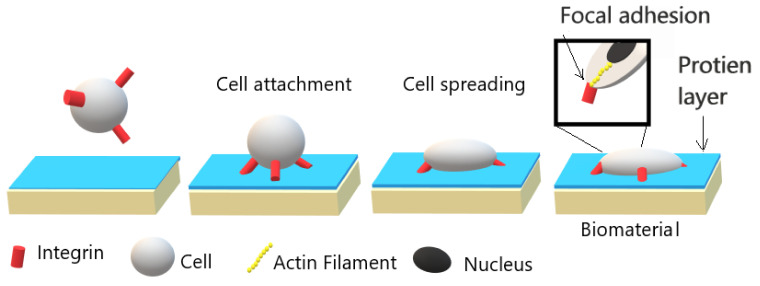
Illustration of cell-biomaterial interaction and its relationship with the strength of adhesion. Adapted with permission from ref. [6]. Copyright 2005 Elsevier.

In cell-material interaction, the initial and most essential event is protein adsorption. The adsorbed protein layer composition and structure depend on the material surface chemical and physical properties; and determine the cellular response and adhesion strength as illustrated in Figure 1. The adhesion of cells is initiated by the adhesion of integrins, which are a receptor protein that is located on cell membrane, with adsorbed protein layer. Following this process, the spreading of cells and enhancing their surface contact area are initiated due to the formation of stress fibers, which are filaments of actin. Finally, strong points of attachment “Focal adhesion” are formed, and the cells are strongly attached to the biomaterial surface. In order to induce cell adhesion, a moderately wettable material surface is preferred [6,7,8]. However, polymer’s surfaces are mostly hydrophobic with low surface energy. In this regard, surface modification of polymers is needed to form a cell-biomaterial bonding surface.

Several methods for surface modification to enhance cell-polymer interaction have been reported in the literature; including physio-chemical, mechanical, and biological treatments [9]. For instance, Suggs [10] used Kr-F excimer laser, which is one of the physio-chemical surface treatments, to modify polymethylmethacrylate (PMMA), polyethylene (PE), terephthalate (PETG), and polytetrafluoroethylene (PTEF) surfaces. The observation was that a high level of UV radiation was sufficient to cause surface damage on PMMA and PTEF; while low radiation dose increased surface roughness of treated polymers, which resulted in increased cell adhesion on PMMA, PETG, and PTEF surfaces. Moreover, UV surface modification was investigated by Olbrich and coworkers [11]. A nanocomposite surface for human umbilical vein endothelial cell (HUVEC) cultivation was modified. It was shown that the formation of N and O functional groups have increased cell growth significantly. Svorcik et al. [12] grafted carbon nanoparticles (CNPs) onto a polyethylene terephthalate (PET) and high-density polyethylene (HDPE) surfaces previously treated with argon plasma. Adhesion and proliferation of vascular smooth muscle cells (VSMC) showed a positive effect for surface CNPs grafting.

Any type of surface modification techniques will affect surface chemistry and morphology, which will modify the substrate mechanical, optical, adhesive, electrical, and morphological properties. These changes should occur in small depth, while the bulk properties remain unaltered [13]. During the last years, atmospheric pressure plasma treatment has been confirmed to be an effective technique for modification of polymer’s surface, especially polymers that are utilized in biomedical applications. The main goal of atmospheric plasma treatment generally is the formation of functional groups on the surface of the polymer. Consequently, polymer’s surface properties including wettability, biocompatibility, and surface chemistry are enhanced. In this technique, only surface properties are changed, the bulk properties of the polymers remain unaffected. Furthermore, it is a solvent free technique that has the ability to modify complex-shaped surfaces. Several methods have been reported in the literature to generate atmospheric pressure plasma according to excitation energy including corona discharge [14], dielectric barrier discharge [15], and atmospheric pressure plasma jet [16]. Several research groups investigated the atmospheric pressure plasma surface modification of polymers and its influence on cell cultivation. For instance, Poly (l-lactic acid) surface modification with corona discharge has been evaluated by Dolci et al. [17]. Fibroblast cells ware cultivated on plasma treated surface, in order to evaluate the surface treatment technique. The results showed that the fibroblasts morphology that adhered on the treated surface was enhanced. Moreover, dielectric barrier discharge plasma treatment of PCL scaffold surface was investigated by Yildrem et al. [18]. The cultivation of osteoblasts on plasma treated surface enhanced the initial attachment, proliferation, and migration of cells.

In this review, we focus on atmospheric pressure plasma treatment of polymeric materials surface and its effect on different cell line cultivation. In the beginning, a brief overview of plasma generation mechanism and the difference between thermal and non-thermal plasma have been mentioned. Furthermore, atmospheric pressure plasma classifications have been discussed in detail, in addition to the plasma gas active species. The second section in this review, describes different modifications of atmospheric pressure plasma treatment on polymer surface including removal of contamination, etching, introducing functional groups, and grafting. The following section demonstrates the cell-biomaterial interaction mechanism and its relationship with biomaterial surface physical and chemical properties. In this section, several studies regarding the interaction of different types of cell lines, including fibroblast, osteoblast, osteosarcoma, endothelial cells, cardiomyocytes, and mesenchymal stem cells with atmospheric pressure plasma treated polymers, have been discussed and summarized. Finally, the challenges and future aspects from our point of view, in addition to the conclusion are mentioned. 

## 2. Atmospheric Pressure Plasma Technology

Plasma is usually referred to as the fourth state of matter. The “plasma” concept was first introduced by the scientist Irving Langmuir in 1928. It consists of atoms, molecules, ions, which are atoms with some removed orbital electron, electrons, and radicals. Plasma can be artificially generated by applying energy to a gas in order to produce ionized gaseous substances containing positive ions and negative electrons. Therefore, from a macroscopic point of view plasma is electrically neutral, even though it contains free charge carrier and it is electrically conductive, so generally plasma exhibits “quasi-neutral” Behavior [19]. The energy that is used to generate plasma can be thermal energy or carried out by applying electrical field, magnetic field or even nuclear reaction [20].

### 2.1. Plasma Generation

**Figure 2:**
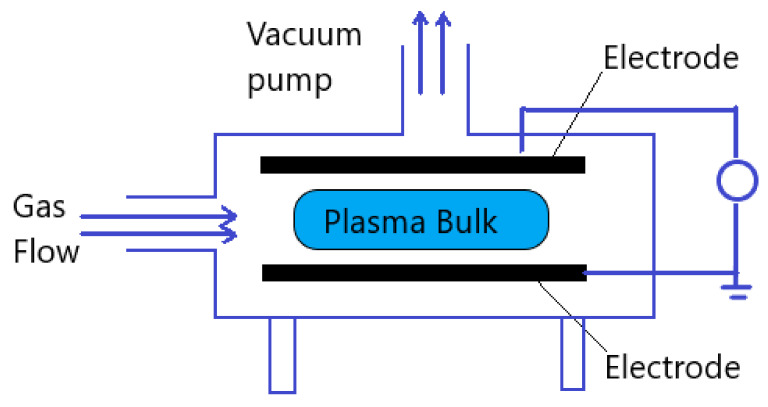
Schematic diagram of general plasma generator.

The atmospheric pressure plasma, described in this review, is generated by applying electrical field to a neutral gas. Either direct current excitation or alternating current excitation, at a frequency range that lies from low frequencies to several GHz, are applied to a gas at atmospheric pressure. The selected plasma gas or gases mixture is generally performed between two plates for plasma generation. Three major reactions that take place in plasma generation are excitation, ionization, and dissociation. Any volume of neutral gas contains few charge carriers, which are electrons and ions. After applying the electrical field to the plasma gas these free charge carriers are accelerated and may collide with atoms in the gas or with the surface of the electrodes. Therefore, the excitation process increases the translational energy of the gas atoms, and transitions internal energy to higher state. If sufficient energy is applied in excitation process, the most loosely bound electrons are removed from the atom, which is ionization. Both excitation and ionization may be due to the reaction by electron collision, ion collision, neutral particle collision, and radiation. On the other hand, dissociation is a result of an inelastic collision of a molecule with an electron, ion, or photon [21]. Generally, a vacuum pump is applied to the plasma generator chamber in order to generate plasma, but in atmospheric pressure plasma generation no need for vacuumed chamber. Figure 2 shows the general plasma generator scheme. 

The plasma can be classified into two groups according to the temperature of electrons or thermodynamic equilibrium; which are:

- Local thermodynamic equilibrium (LTE) (or thermal) plasma, and
- Non-local thermodynamic equilibrium (non-LET) (or non-thermal) plasma.

The LET or thermal plasma is composed of electrons, ions, and neutrals in thermal equilibrium with each other, and background gas is heated up to a few thousands of Kelvin degrees. On the other hand, the non-LET or non-thermal plasma temperature of electrons, ions, and neutrals are quite different, generally, the electron temperature is at a much higher temperature than other particles. The background gas temperature in non-thermal plasma is quite low. Therefore, the discharge energy mostly goes into the production of energetic electrons. Owing to the low temperature of non-thermal plasma, it is capable to treat thermolabile materials with this plasma. Moreover, non-thermal plasma is easily adapted to complex geometric. Therefore, it is widely used in material surface modification, medical sterilization, and microbial decontamination applications [22]. 

Plasma is generated under different gas pressure. In the past several decades, atmospheric pressure plasma has been explored to avoid the expensive and complicated vacuum system. As described above, atmospheric pressure plasma can be classified as thermal and non-thermal plasma [23]. Mostly, in polymeric material surface treatment application non-thermal plasma has been utilized, also it is generated by applying electrical field at atmospheric pressure.

### 2.2. Classification

Atmospheric pressure plasma is an economical and easy-to-use technology utilized in many applications including surface modification. Several methods have been reported in the literature to generate plasma at atmospheric pressure. In general, these methods can be classified according to excitation frequency as follows:

- DC and low-frequency discharges such as corona discharges and dielectric barrier discharges,
- Radiofrequency discharges such as atmospheric pressure plasma jets, and
- Microwave induced plasmas.

The DC and low-frequency discharge can work either with a continuous or a pulsed mode depending on their design. The continuous working mode; for example, the arc plasma torches, are mostly thermal plasma. On the other hand, the pulsed mode; such as corona and dielectric barrier discharges (DBD), are generally non-thermal treatment which is suitable for polymer surface treatment. 

The radiofrequency discharge is generated by applying a radiofrequency electrical field on a flow of gas or a mixture of gases. Regarding their structure, the radio frequency discharge can be classified as capacitive or inductive coupled. The capacitively coupled plasma discharge (CCP) is a low power discharge; in contrast, the inductively coupled plasma (ICP) is a high-powered discharge working in the kW range [24]. 

**Figure 3:**
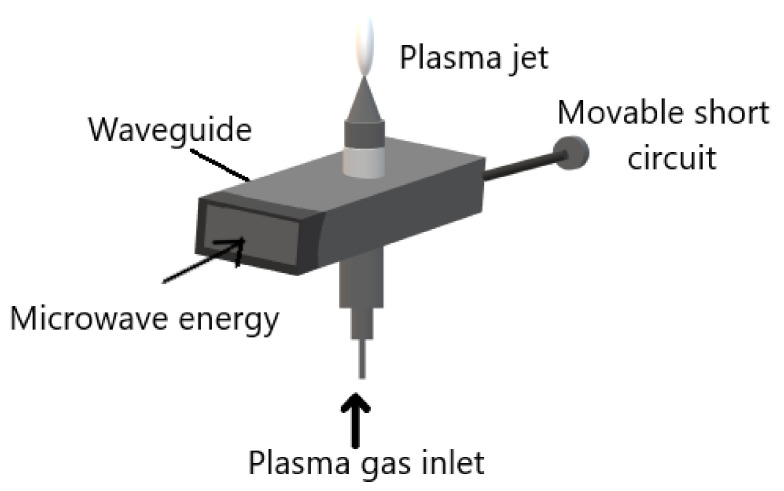
Schematic diagram of microwave induced plasma system. Adapted with permission from ref. [26]. Copyright 1984 IOP Publishing LTD.

The microwave induced plasmas (MIPs) are electrode-less systems. All the MIPs work under the same principle that is microwaves guided along the system, and the energy is transmitted to plasma gas. Several elastic collisions between plasma gas electrons and heavy particles occur. Therefore, the electron gets enough energy to produce inelastic collisions and the gas is partially ionized and becomes plasma. All the MIPs consist of:

- A microwave power source;
- microwave equipment for example waveguide;
- an ignition system; and
- gas injection system. As shown in Figure 3.

The microwaves are generated using a magnetron at a frequency of a few GHz. It is guided to the system by a waveguide or coaxial cable. The gas temperature of the plasma can vary from room temperature to few thousand degrees. Microwave plasma torch is an example of MIPs which creates plasma that flows in the air. The electrodes-free system allows it to be used as a torch which is easily used and applied. Furthermore, the ease of ignition utilizing any gas or gas mixture is also another advantage of the MIP system [25].

Although different atmospheric pressure plasma sources have been studied through the years for surface modification applications, the vast majority of studies are dealing with corona discharges, dielectric barrier discharges, and atmospheric pressure plasma jet. Therefore, here we discuss these plasma sources in detail, mentioning their basic configurations, applications, advantages, and disadvantage. 

#### 2.2.1. Corona Discharges

**Figure 4:**
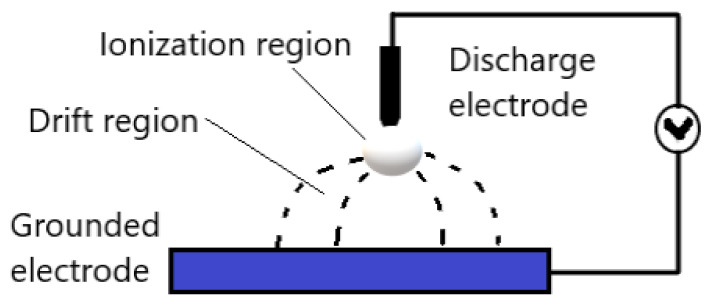
Schematic diagram of general point-to-plane corona discharge. Adapted with permission from ref. [36]. Copyright 1969 Elsevier (Open Access).

Corona discharge generates plasma on a sharp point, edge, or thin wire, under atmospheric pressure with mostly air as a plasma gas. The corona discharge is spatially non-uniform, and the ionization and the electrical field mostly are located near the pin-shaped electrode. The most popular configuration of corona discharge is the point-to-plane, whereas the strong electrical field generated between the sharp point and the flat electrode results in ionization of the gas plasma. Figure 4 demonstrates the point-to-plane corona discharge general configuration. However, the most popular corona discharge configurations in industrial applications are the wire-to-cylinder, wire-to-plane, and saw-to-blade [27]. The reasons for that are the homogeneous distribution of plasma discharges, and the ease of implementation. The sharp electrode in corona discharges is either positive or negative and accordingly the discharge is called positive or negative corona, respectively [28].

Although corona discharge is a versatile and environmentally friendly technique for surface treatment and ozone generation, it is still not gained much commercial momentum due to some disadvantages with such discharge such as non-uniformity and power loss. Furthermore, corona discharge volume is very small and due to that, the treatment surface is very small [29]. Because of the corona discharge advantages, including the ease of implementation, it has numerous applications for example, removal of unwanted volatile organics from the atmosphere [30], material’s surface treatment [31], gaseous contamination treatment [32], water purification [33], and ozone production [34]. Furthermore, the negative corona discharge have been studied for the possible use for treatment of microscopic cavities in dentistry due to its antibacterial effect and hairline geometry [35].

#### 2.2.2. Dielectric Barrier Discharges

**Figure 5:**
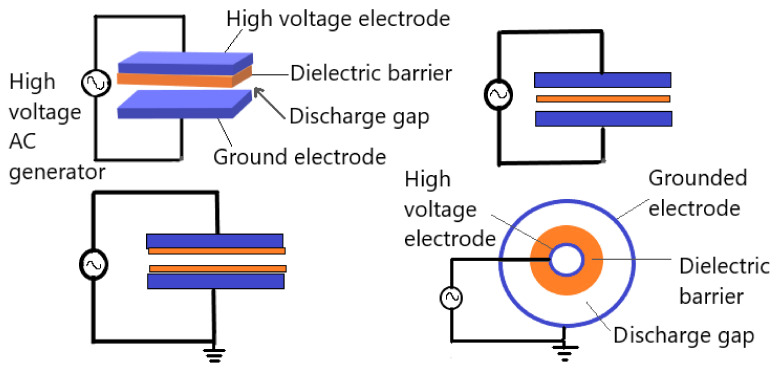
General planer and cylindrical dielectric barrier discharge geometrical configurations. Adapted with permission from ref. [45]. Copyright 2003 Elsevier.

The dielectric barrier discharge (DBD) is plasma established in a gas gap between different electrodes configurations, with an insulating material between the electrodes. Typically, the insulating materials “barrier” such as glass, quartz, ceramics, plastics, and silicon rubber are used. The classical configurations of DBDs are either planar or cylindrical with at least one of the electrodes is covered with dielectric layer, typical electrodes arrangements are shown in Figure 5. Furthermore, several novel configurations of DBDs have been developed in order to adapt with many different applications, including sliding discharge, capillary plasma electrode discharge, microcavity plasma array, and coplanar configurations [37]. Plasma in DBDs is generated by applying high voltages; in the range of some kV. In order to achieve stable performance, the alternating current frequency range from low frequencies up to the order of several MHz. The gap between the electrodes is usually filled with gas or a mixture of gases and can vary from a few micrometers to some centimeters [37,38,39,40]. DBD usually exhibits two different modes depending on electrode geometries and setups; which are Filamentary and diffuse discharges. The filamentary mode is non-uniform discharges with many small discharge channels along the electrode called microdischarges. However, the filamentary DBD appears to be uniform to the naked eye or slow cameras after a long duration treatment or if the number density of microdischarges per cycle is high [41,42,43]. On the other hand, DBD diffuse mode is a uniform and homogeneous discharge [39]. Generally, the filamentary mode are utilized in DBD applications, because the diffuse mode operates under special conditions [44,45]. 

The DBDs have many advantages, including low energy consumption contrary to corona discharges, low operational cost, scalability, effectiveness, and short processing time. Whereas, the main drawbacks are the high ignition voltage, and limited discharge gap height that is directly related to plasma homogeneity [46]. Many applications of DBD plasma, according to its advantages, are available such as ozone generation, excitation of CO_2_ lasers and excimer lamp, wastewater purification, plasma chemical vapor deposition, and surface modifications. Furthermore, DBD plasma has a huge antimicrobial potential, therefore it is used to improve food safety and extend the food shelf life [47]. Moreover, DBD has been widely used in medical applications including cancer treatment, dentistry, and regenerative medicine [48]. 

#### 2.2.3. Atmospheric Pressure Plasma Jets

The atmospheric plasma jet (APPJ) is a plasma that is generated in non-sealed electrode arrangement and projected outside the electrode arrangement into the environment. The unique feature of APPJ resides in the fact that the plasma is generated within the device and extends in the open space in the form of “plasma plume”. Many different APPJ devices have been developed so far, differing in the electrode’s arrangement, strategy exploited for plasma generation, and shape and dimension of plasma plume. Generally, almost all the plasma jets generated within a duct through which the plasma gas or gases mixture flows and propagates. Furthermore, the plasma propagates through the duct and ejected outside the device, along the gas flow direction. The APPJ can be classified according to the electrode configuration and power supplies into corona plasma jet, DBD-based plasma jet, radiofrequency (RF)-generated plasma jet, and microwave (MW)-driven plasma jet. 

**Figure 6:**
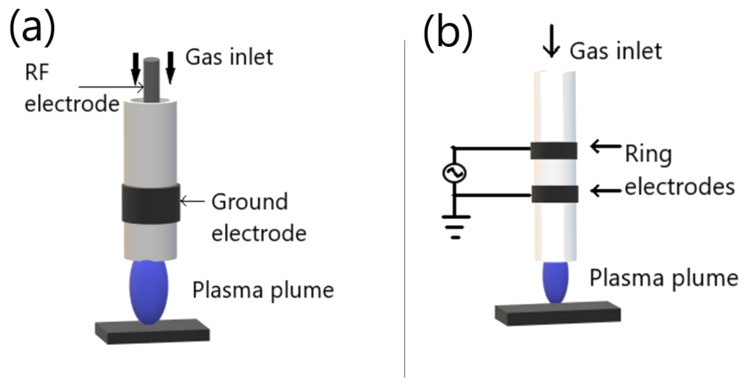
Schematic diagram of general APPJ: (**a**) RF-generated plasma jet; (**b**) DBD-based plasma jet. Adapted with permission from ref. [51]. Copyright 2017 Elsevier.

The RF-generated plasma jet can be capacitively driven or inductively driven, and usually operate in the frequency range 1–100 MHz. The RF-generated plasma jet general capacitive configuration is reported in Figure 6a. The RF-generated plasma jet operates in RF power, which generates a very uniform and stable discharge between two concentric metallic electrodes. 

On the other hand, the general DBD-based plasma jet is shown in Figure 6b, two ring electrodes are coaxially arranged around a dielectric tube. The application of gas flow is parallel to the electrical field generated in the ring electrodes. Different parameters control the propagation of plasma plume from the device exist, including the electrical field geometry, the gas flow conditions, and the source design [49,50,51]. 

In addition to the advantages of the atmospheric pressure plasma including manageability and cost-effectiveness, the APPJ small plasma dimensions, penetrability, and distinctive remote operation, give the ability for plasma jet to treat materials with complex geometries and micro-structured pores. Nevertheless, the main drawback of APPJ is the small spot size which makes it inappropriate for homogeneous treatment of large surfaces [52]. The APPJ has received a significant attention, due to its advantages, in various fields of science and technology. For instance, deposition of coating, generation of nanoparticles, surface modification, and biomedical applications are some of APPJ applications [53]. As an example, Bernhardt and coauthors [54] have showed that different APPJs could be a promising and inexpensive treatments for many diseases, including atopic eczema, itch, pain relief, and epidermal barrier defects. Whereas APPJ already reached standard medical care status for wound healing and scar treatment. 

### 2.3. Active Species 

Plasma treatment is a widely used technique to modify polymeric surfaces. Argon (Ar), helium (He), nitrogen (N_2_), and oxygen (O_2_) are mainly the gases used in plasma generation for polymeric surface modification. The interaction between the active species in plasma generation using these gases and the polymer’s surface leads to the formation of free radicals at the surface of polymers [55]. For instance, using oxygen as plasma gas leads to a functional group containing-oxygen atom, for example, hydroxyl group, carbonyl group, and in some cases carboxylic group. A different functional group can be introduced into the surface of polymers depends on the gas or gases mixture used in plasma generation [7]. A list of plasma gases and their applications is listed in Table 1.

Generally, plasma gases are classified into five different types, which are:

- Oxidizing gases such as O_2_, air, N_2_O, and H_2_O;
- Reducing gases such as H_2;_
- Nitrogen containing gases such as NH_3_,
- Fluorine containing gases such as CF_4_ and SF_6_, and
- Polymerizing gases which use monomer gases to direct polymerize or graft a layer onto a substrate.

Each type of plasma gases has different application, especially in surface modification. For instance, oxidizing gases are utilized in leaving oxygen species on polymer surface or removal of organic contamination by oxidation, while reducing gases provide replacement of F or O in surface. On the other hand, nitrogen containing gases are used to generate amino group on substrate surface, which improve biocompatibility, wettability, and bondability of substrate. Whereas fluorine containing gases provide surface etching and plasma polymerization which make surface inert and hydrophobic [56,57].

## 3. Modification of Polymeric Surfaces by Atmospheric Pressure Plasma

Polymers derived from either natural or synthetic sources and represent the most used and investigated class of biomaterials in biomedical applications. This versatility is attributed to the wide spectrum of physical and chemical properties, ease of fabrication with a wide variety of structures that range from simple mats to complex shapes, and biocompatibility. There are many biomedical applications utilizing polymers, for instance, drug delivery vehicles [58,59], tissue engineering scaffolds [60,61], wound dressing [62,63,64], and biomedical sensors [65,66]. Although polymers have suitable bulk properties for some biomedical applications, their surface properties are not appropriate. The surface properties including surface morphology, surface energy, ionic species, surface chemistry, and water content can be modified using many techniques [67]. Three major classes of surface modification can be considered. These are surface modification by a chemical agent, surface modification by a physical agent, and the immobilization of biological compounds or cells on material’s surface. The modification of the surface by a chemical agent, for example, surface coating, chemical grafting, and surface oxidation leads to incorporation of functional group, including carboxylic acid group, hydroxyl group, and amino group, on polymer surface. Flame treatment, ultraviolet radiation exposure, laser ablation treatment, and X-ray treatment are examples of physical surface modification techniques [68]. Furthermore, surface modification techniques can range from large-scale treatments such as ultraviolet radiation exposure, to precise ones such as laser ablation. Any surface treatment technique that changes the chemistry or morphology of the substrate surface, will affect the mechanical, electrical, tribological and many other properties of the treated substrate. However, these changes should be limited to small depth from the surface, therefore the bulk properties remain unchanged [69]. 

In plasma surface modification, the polymeric materials undergo certain changes due to the interaction of the polymer surface with different plasma species. This interaction can cause alterations in physical nature as well as surface chemistry. Furthermore, the morphology and roughness of polymer’s surface can be modulated in order to achieve the suitable interaction between the polymer and biological environment. Plasma surface modification mostly achieved using gases such as air, oxygen, nitrogen, argon, and helium. Several surface modification techniques can be attained using plasma treatment including removal of surface contamination, etching, and substitution of a chemical group [70]. The various effects of plasma surface treatment on polymers are not limited to surface modifications. Plasma-induced grafting and plasma polymerization also processes that treat polymer surface using atmospheric pressure plasma. However, they differ from surface modifications by introducing monomers into treated surface [71]. The main difference between plasma-induced grafting and plasma polymerization is that plasma polymerization is the formation of ultrafine layer of polymeric material without covalently binding with the modified polymer’s species, while plasma-induced grafting is the formation of grafted layer of monomer on the polymer surface [72]. Here, we present general information about different polymer surface modifications. 

### 3.1. Removal of Surface Contamination

**Figure 7:**
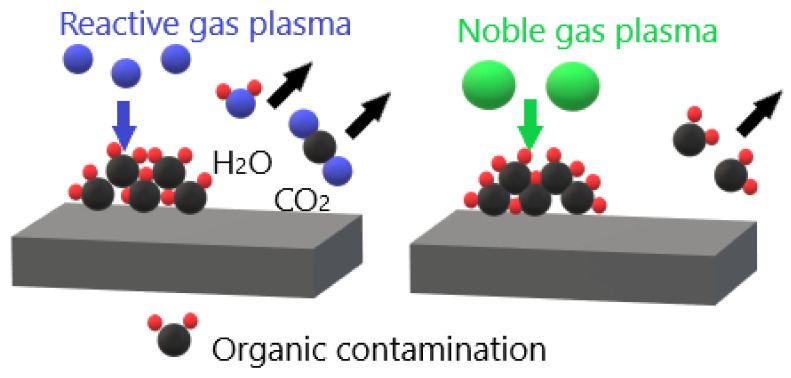
Removal of surface organic contamination with atmospheric pressure plasma generated with reactive gas (chemical decontamination) and noble gas (physical decontamination).

Surface contamination sources including air pollutants or fingerprints can be attenuated using plasma treatment. Moreover, various organic contaminations are removed using plasma treatment [73]. Contamination of surfaces with organic contamination is the major source of problems in biomedical application, especially contamination with biofilm which is the most common source of microbial contamination. Therefore, the complete removal of organic contamination is mandatory in biomedical application. The highly reactive agents in atmospheric pressure plasma have a high potential in microbial decontamination [74]. For instance, in case noble gas such as argon is the plasma gas, surface decontamination mostly achieved by plasma physical etching [75]. On the other hand, if reactive gas, such as oxygen or nitrogen, is used to generate plasma, oxidation or reduction of contamination is mainly responsible for the surface decontamination. The main effect of noble gas plasma is physical etching by ion bombardment, while mostly the reactive gas plasma produces chemically active species that oxidize the organic contamination with the release of CO_2_ and H_2_O as shown in Figure 7 [76]. 

Plasma surface decontamination offers a short duration at low-temperature decontamination process which is suitable for a wide range of materials including polymers. For instance, microwave-induced plasma reported a high sterilization efficiency due to the high concentration of oxygen [77]. The atmospheric pressure plasma effect on the decontamination of surface has been investigated widely over the past years. They have concluded that the reactive agents in plasma including oxygen species were effective in microbial decontamination. However, the complete removal of residual dead organic contamination is a challenging task. In this regard, atmospheric pressure plasma treatment manifests a promising technique to decontaminate polymer surface and eliminate the residual dead organic materials [74].

### 3.2. Etching

Plasma etching occurs under specific plasma conditions, which leads to surface ablation. Due to this, the surface morphology is altered, and the surface roughness is increased, which leads to increase surface-to-volume ratio [78]. In plasma surface modification of polymers, the plasma etching depends on the power and duration of plasma treatment. Furthermore, the physical and chemical properties of the polymer play crucial role in plasma etching. For instance, the rate of etching of polymer surface depends on the degree of crystallinity of the polymer. Therefore, the polymer with higher degree of crystallinity shows lower etching rate, than the less crystalline (more amorphous) polymer, that is because the structural integrity of crystalline polymer [79]. Moreover, the polymer molecular weight affects the rate of etching [80].

### 3.3. Substitution of Functional Group

The functionalization of polymer surface by applying atmospheric pressure plasma can be categorized according to its functions, such as hydrophilization, hydrophobization, adhesability, printability, and paintability. Moreover, it can be categorized according to chemistry of the process into oxidation, nitration, and fluorination [70]. Atmospheric pressure plasma treatment has been widely used to modify polymer surface, thereby improving its surface energy, wettability, adhesion, and biocompatibility, by substitution of functional group on polymer surface [13,81]. For instance, the substitution of atoms including oxygen and nitrogen into polymer surface during atmospheric pressure plasma treatment leads to the formation of function group, such as hydroxyl, carbonyl, carboxylic, and amino groups, that influence the surface energy. 

## 4. Cell Cultivation Verification of Atmospheric Pressure Plasma Treatment

Atmospheric pressure plasma treatment is a convenient technique to modify the polymeric surface and enhance the interaction between biomaterial and cells around it. Once the biomaterial is implanted inside a biological environment, several reactions are established between the biomaterial, specifically the biomaterial surface, and the host tissue. These reactions mostly can be classified as normal reactions including local reactions (such as inflammation and infection) and systematic reactions (such as hypersensitivity). However, if these reactions are not controlled properly, they may lead to implant failure, and in the worst-case host death [82].

**Figure 8:**
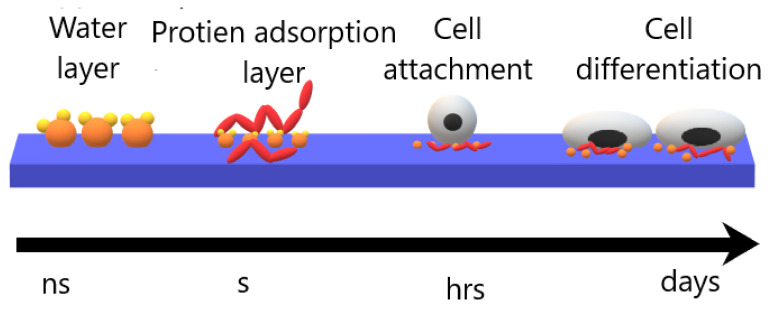
Cell-biomaterial interaction stages.

In order to understand and avoid such effects, it is important to understand the cell-biomaterial interaction. When a biomaterial is placed in a living organism four main stages occur, which last as soon as the biomaterial is implanted up to decades. The first step starts within a nanosecond, a water shell around the biomaterial is created. In the next step, a layer of adsorbed protein covers the biomaterial in few hours [83]. This adsorbed protein layer, such as fibronectin and vitronectin, contains one of the important cell receptors called “integrins”, and present mainly in the extracellular matrix (ECM). The third stage occurs once the cells from the surrounding tissue interact with the adsorbed protein layer. Cells sense surroundings through “protrusions”, which is a micrometer actin filament mesh. These protrusions extend up to a thin actin filament bundle called “filopodia” that sense the ECM. When the filopodia find an integrin binding site, a feedback signal allows the cell to adhere to that region. This stage may last from hours to days, after the adhesion of cells to the biomaterial, spreading, differentiation, and migration of cells take place. Moreover, this stage depends on the biomaterial surface’s properties, biophysical environment, and biological molecules. The last stage determines the life of the implanted biomaterials which can last several decades [84,85,86]. Figure 8 demonstrate the cell-biomaterial interaction stages.

Since the adsorbed protein layer on the biomaterial determines the response of the surrounding cells. Furthermore, the material’s surface properties including surface energy, surface chemistry, ionic species, topography, cleanliness, crystallinity, water content, and protein denaturation tendency determine the activity of the adsorbed protein layer. Therefore, biomaterial’s surface properties are the most essential properties in the interaction with the biological environment. Since polymers have inappropriate surface properties, modification of the polymer’s surface is needed. Many traditional surface modification techniques have been investigated, but in this review, we are discussing atmospheric pressure plasma treatment particularly. Surface modification with atmospheric pressure plasma introduce various functional group including hydroxyl group, amino group, and carboxyl group; or graft hydrophilic polymers on polymer’s surface thereby, the surface becomes hydrophilic which is favored by cells [87,88,89,90]. In the next part, we will focus on different types of cell lines that interact with surface-modified polymers by atmospheric pressure plasma treatment.

### 4.1. Fibroblasts

**Figure 9:**
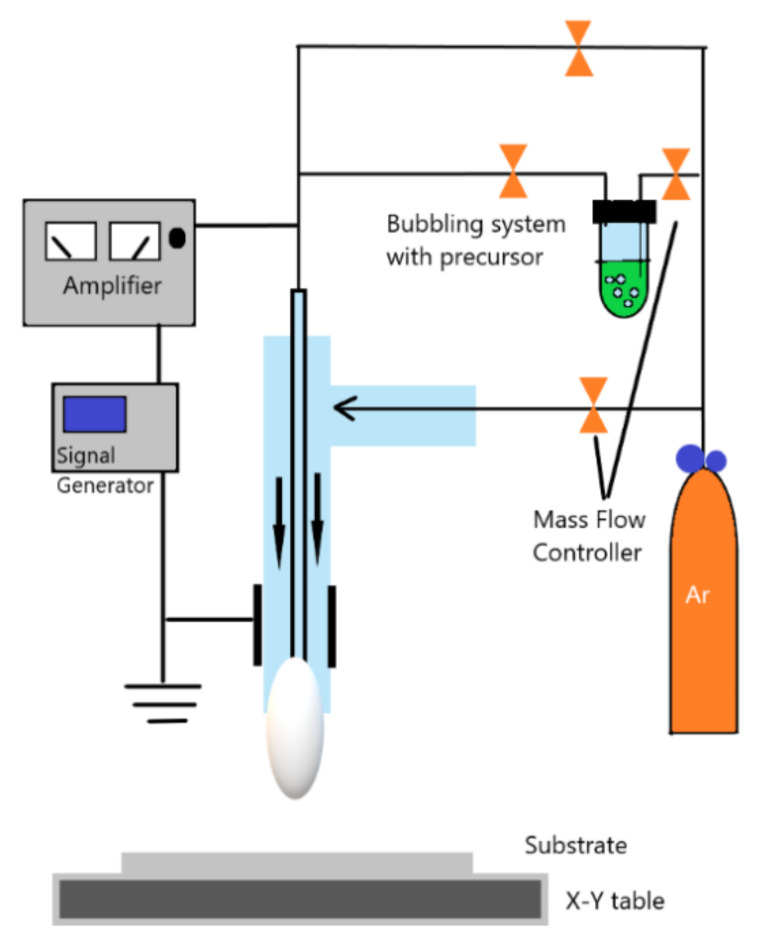
Schematic diagram of the APPJ system. Adapted with permission from ref. [94]. Copyright 1970 IOP Publishing LTD.

It was widely reported that fibroblasts were utilized in the investigation of surface biocompatibility of biomaterials after modifications [91,92]. One of this review authors investigated the plasma surface treatment intensively. Starting with low-pressure water/oxygen plasma treatment of PCL and non-woven polyester NWPE fabric discs in 2011. The polymers surface modification was investigated using periodontal ligament fibroblasts cells. Fibroblasts cells spreading, viability and growth were improved as showed by the workers [93]. Moreover, the deposition of PCL homopolymer and poly ε-caprolactone-polyethylene glycol (PCL-PEG) copolymer onto electrospun PCL scaffold by APPJ was investigated [94]. A schematic diagram of the system used for surface modification and polymerization is illustrated in Figure 9. Polymer deposition scaffold showed better stability and higher hydrophilicity even when compared to the scaffold treated with APPJ in Argon alone. Furthermore, XPS analysis showed C1s% composition decreased and O1s% content increased for the deposited scaffold compared to untreated one. Cell culture was carried out using the L929 fibroblasts cell line and showed promising cell adhesion, proliferation and growth enhancement for the deposited PCL-PEG scaffold specifically.

Furthermore, PCL/Chitosan surface treatment by atmospheric pressure plasma was intensively investigated by the same author and team. In the first study, atmospheric pressure DBD was applied to the PCL/Chitosan/PCL layer-by-layer 3D structure. Two different plasma gas mixtures, Ar/O_2_ and Ar/N_2_ were used, in order to investigate the topographical change and the hydroxyl and amino functionality, respectively. The cell culturing results using the L929 fibroblast cell line showed no significant changes for the Ar/N_2_ surface treatment. On the contrary, surface modification with Ar/O_2_ plasma caused increase cell attachment, viability, and proliferation, due to alteration in topographical and oxygen-containing functionality [95]. In the second investigation, two different atmospheric pressure surface treatments, DBD and plasma jet, and three different plasma gases, Ar/O_2_ and Ar/N_2_ for DBD and dry air for plasma jet were applied on the same PCL/Chitosan scaffold. Optimization of both plasma parameters was obtained using contact angle measurement; and SEM and XPS were utilized to determine the topographical and functional changes of the surface, respectively. Human fibroblast (MRC5) cell culture studies were carried out, it was shown that treatments with Ar/O_2_ and dry air plasma jet enhanced immediate cell attachment, and seven-day cell viability, attachment and growth. The reasons for that are, the increased wettability, surface roughness as well as functionality of OH and NH_2_ in dry air plasma jet, and OH in DBD Ar/O_2_ plasma treatment [96]. The PCL substrate surface treatment was investigated by Han and coworkers [97] by using Ar atmospheric pressure glow discharge APGD. Cell cultivation studies utilizing neonatal human dermal fibroblast (nHDF) were carried out. Results showed that 4 h seeding cell attachment density increased 60-fold on 1 min treatment, and more than one hundred-fold on 10 min treatment over untreated PCL. Besides, the rate of cell proliferation and cell attachment was higher in treated PCL than untreated one.

**Figure 10:**
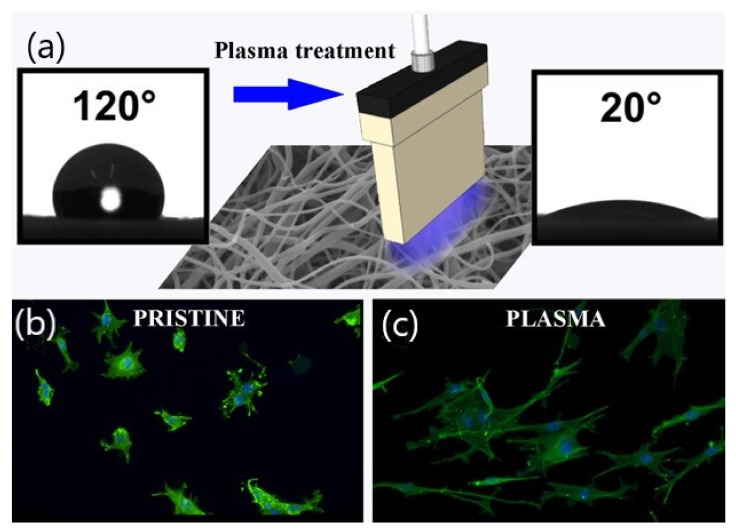
Illustration of PLLA surface modification utilizing LCD and fibroblast morphology of treated and untreated substrate; (**a**) plasma treatment of electrospun surface and contact angle of pristine and plasma treated surface; (**b**) small, rounded, and star-like fibroblasts with short processes attached on pristine surface; (**c**) elongated with dendritic morphology fibroblasts attached on plasma treated surface. Reprinted with permission from ref. [17]. Copyright 2014 John Wiley and Sons.

Fibroblast cell cultivation of atmospheric pressure plasma surface modification of polyethylene and polylactic acid was investigated by many research groups. For example, Pandiyaraj et al. [98] investigated the effect of oxygen flow rate in atmospheric pressure argon plasma treatment of low-density polyethylene (LDPE). In order to investigate the morphology, hydrophilicity, and surface chemistry; atomic force microscopy (AFM), contact angle, and X-ray photoelectron spectroscopy have been utilized, respectively. The modified surface cytocompatibility was analyzed by NIH 3T3 fibroblast and showed improvement in adhesion and cytocompatibility of cells, due to the incorporation of a polar functional group. Poly (l-lactic acid) (PLLA) linear corona discharge (LCD) surface modification was evaluated using different characterization techniques by Dolci and coworkers [17]. Mouse embryonic fibroblast (MEF) was cultured to evaluate the surface treatment technique, and the results were analyzed by computerized morphometry. It was founded that the fibroblast seeded on untreated PLLA were small, rounded, and star-like with short processes, while cells on the treated scaffold were elongated with dendritic morphology as illustrated in Figure 10. Therefore, the plasma treated PLLA showed a higher field/body area ratio and reduced percentage of apoptotic nuclei. 

Polystyrene and Teflon surface modification techniques were widely investigated due to their natural hydrophobicity which is not favored in biomedical applications [99,100,101]. Sramkova and coworkers [102] investigated the post-treatment of poly(2-oxazoline) (PO_x_) deposited on polytetrafluoroethylene (PTFE) substrate. The resulting copolymer PMEO_x_ was treated with air and argon diffuse coplanar surface barrier discharge (DCSBD), and 3T3 mouse fibroblast was seeded to investigate cell adhesion. Cell adhesion enhancement for the DCSBE treated copolymer was found the best among PTFE and copolymer without treatment. Polystyrene (PS) plate air APPJ surface modification was investigated by Lee et al. [103], and L929 mouse fibroblast was utilized for cell cultivation characterization. Actine filaments, vinculin, and nuclei were visualized using a special adhesion kit, and the protein tyrosine kinase 2 (PTK2) was evaluated. The results showed an increase in the number of the attached fibroblasts, also the spreading phenotype of the treated PS plate was wider which can be considered as positive indication of improvement in immobilization and attachment of fibroblast. In addition to that, the vinculin protein and PTK2 increased on the treated PS plate which are considered as adhesion proteins and gene expression for the formation of focal adhesion. Table 1 Summarizes the previous studies on atmospheric pressure plasma treatment of polymers and the influence on fibroblast cell line. 

### 4.2. Osteoblast and Osteosarcoma

In tissue engineering scaffolding, the more biocompatible the surface of the scaffold, the more likely it provides a 3D framework for bone cells to attach and develop. Polymers generally have favorable bulk properties for hard tissue scaffolding [104]. However, various disadvantages such as hydrophilicity and biocompatibility limited their application. In order to overcome these disadvantages several surface treatments techniques have been applied to polymer surface. One of these treatments is atmospheric pressure plasma treatment. Several studies, regarding bone cell cultivation to characterize polymer atmospheric pressure plasma surface treatment, were found in the literature. 

**Figure 11:**
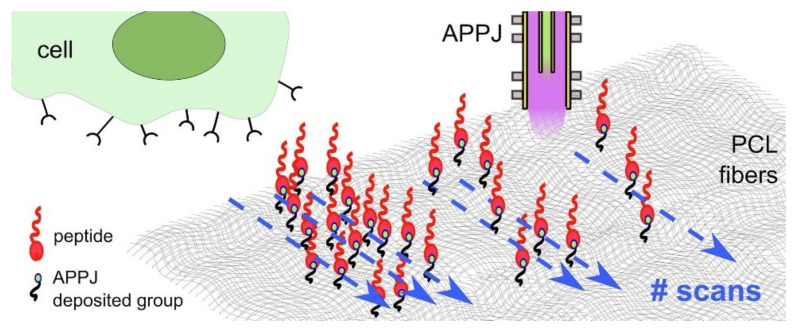
Schematic diagram of the sample preparation process. Reprinted with permission from ref. [114]. Copyright 2020 Elsevier.

PCL is an aliphatic polyester with promising potential in biomedical applications especially bone tissue engineering and drug delivery applications due to its bulk properties including mechanical and structural properties [105,106,107,108,109]. However, PCL surface physiochemical properties are not adequate for cell attachment due to low surface energy and hydrophobicity. The PCL surface modification can be modified using several techniques including plasma treatment [110,111,112]. Many research groups discussed PCL surface treatment using atmospheric pressure plasma and enhancement of cell-biomaterial interaction using osteoblast or osteosarcoma cells. Yildrem and coworkers [18] used DBD plasma to improve 7F2 mouse osteoblast cell proliferation and adhesion on PCL scaffold. The results showed that DBD oxygen plasma treatment not only enhances the hydrophilicity and surface energy of PCL but also improves the initial attachment, proliferation and migration of osteoblast. Trizo et al. [113] work was about surface modification of porous PCL scaffold utilizing of He/O_2_ APPJ intending to improve human Saos-2 osteoblast and scaffold interaction. A comparison between untreated, He treated, and O_2_/He treated scaffolds cells morphology was attained. Consequently, clusterization and the presence of actin stress fibers, which is an indication of cell adhesion, of Saos-2 cell on APPJ treated samples are signs of favorable cell-biomaterial interaction. Maffi et al. [114] utilized a novel technique of selective peptide immobilization on PCL electrospun substrate. The primary amine functionalities NH_2_ coating was deposited to the surface by applying (3-Aminopropyl) triethoxysilane (APTES) using APPJ and by successive selective covalent linking of this amine with synthetic human vitronectin adhesion cue (HVP) as demonstrated in Figure 11. Different surface coverage of plasma coating was achieved, which in turn led to a diverse amount of linked HVP. The cell cultivation with human osteoblast showed improvement in cell viability after HVP functionalization and an increase in cell number was directly correlated with the number of APPJ scans.

Plasma treatment of poly lactic acid was widely investigated [115,116], but here only surface modification with atmospheric plasma is demonstrated. For instance, atmospheric pressure plasma surface modification of poly (l-lactide) was investigated using cell cultivation of MC3T3-E1 cells [117,118]. The result showed that PLLA samples treated in air or CO_2_ gas were significantly superior in number and growth of adhering cells comparing to C_3_F_8_ treated substrate. Furthermore, APPJ surface treatment of Poly (l-lactic acid) PLA substrate for polymerization of dual tripeptide arginine-glycine-aspartic acid (RGD), which is a specific recognition site of integrin of targeted ligand, was investigated. Human bone osteosarcoma (MG-63) cell line was used in cell-biomaterial interaction investigation. MTT, ALP activity and, Western blot assays of gene expression pre-test characterization tests showed that cell proliferation and ALP activity significantly enhanced for APPJ treated substrate even without immobilization of RGD or glucan [115]. Polystyrene (PS) and polyethylene (PE) are widely utilized and investigated as materials used for medical applications [119,120]. Ayan et al. [121] evaluated utilizing atmospheric pressure microplasma jet surface modification on PE. The results demonstrated that surface characterization including surface roughness and concentration of oxygen-containing functional groups have been changed. Consequently, the 7F2 osteoblast cells attached and survived on the treated substrate comparing to the un-treated one. Ultra-high molecular weight polyethylene (UHMWPE) surface modification using He/O_2_ APPJ was investigated by Perni and coworkers [122], in order to enhance the wear performance of UHMWPE without affecting the cytocompatibility. No significant difference in cell adhesion between the treated and untreated substrate was found. A combination of surface modification techniques was attempted by Dowling and coworkers [123] on PS substrate. They applied atmospheric pressure plasma to deposit a siloxane coating, then low-pressure radio frequency plasma to fluorinate the PS substrate. The siloxane coating was obtained using liquid poly (dimethylsiloxane) (PDMS) precursor. Cell cultivation investigation was carried out using MG-63 osteosarcoma cells showed that different surface treatments change surface morphology and hydrophilicity, and consequently cells adhesion. Surface modification with atmospheric pressure plasma and siloxane showed the best cell-biomaterial interaction at ~64° contact angle for coating on a smooth surface and ~57° contact angle for coating on a rough surface. In addition to that Fluorination of the surface decreases cell adhesion. D’Sa et al. [124] investigated the PMMA/PS polymers blend surface modification using DBD plasma treatment. The results of the cultivation of human fetal osteoblast cells (hFOBs) showed that increasing the hydrophilicity/wettability of the surfaces by oxygen functionalization that causes an increase in the cellular response. Table 2 demonstrates an overview of different polymers and atmospheric pressure plasma surface modification techniques and their influence on different bone cells. 

### 4.3. Endothelial 

Vascular tissue engineering is a challenging field due to the difficulty to achieve satisfied physiological, immunological, and manufacturing demands [125,126,127]. Several research groups focused on improving the performance of vascular implants. Polymers including PU, PTFE and PCL are widely used in vascular tissue engineering applications due to their excellent tensile strength, blood compatibility, and bio-durability [127,128,129]. However, these materials suffer from drawbacks, such as thrombogenicity, that can be improved by adding endothelial cell lining on the blood-contacting surface. Therefore, in order to achieve better endothelial cell attachment, surface modification techniques, for example, atmospheric pressure plasma treatment, was applied to polymer’s surface.

PCL electrospun nanofibers scaffold surface was modified with inducting coupled radio-frequency glow discharge (RFGD) and grafting gelatin to improve the biocompatibility with endothelial cells (EC) [130]. The RFGD plasma treatment with air introduced -COOH groups, that enhance the covalent grafting of gelatin molecules using water-soluble carbodiimide as a coupling agent. Cell culturing with human coronary artery endothelial cells (HCAEC) showed that surface modification of PCL enhances the spreading of cells and the formation of pseudopods. Furthermore, immunostaining micrographs indicated that the expression of platelet-endothelial cell adhesion molecule 1 (PECAM-1), intercellular adhesion molecule 1 (ICAM-1), and vascular cell adhesion molecule 1 (VCAM-1) characteristic markers were maintained on the treated scaffold [130]. The PLA-based electrospun surface was also modified with atmospheric pressure plasma in order to assist immobilization of biomolecules. Kudryavtseva et al. [131] modified electrospun PLLA surface utilizing self-sustained volume discharge in atmospheric pressure air and attachment of hyaluronic acid (HA) on the plasma-treated surface. The application of plasma treatment activates the surface that allows for non-destructive immobilization of HA compound. Biocompatibility of scaffold using human umbilical vein endothelial cell (HUVEC) was investigated, it showed that both scaffolds treated with atmospheric pressure plasma have increased cells viability. De et al. [132] compared between three different scaffolds in HCAEC adhesion and attachment under laminar flow. These scaffolds were (1) bare glass substrate; (2) glass substrate coated with polyurethane (PU), with and without helium atmospheric pressure plasma treatment; and (3) collagen-treated PU-coated glass substrate. The results showed that the plasma-treated PU substrate was the best scaffold according to improvement of hydrophilicity, oxidation of the surface, surface roughness, and enhancement of HCAEC growth and adhesion under laminar flow. DBD surface modification with atmospheric pressure discharge was shown by Bilek and coworkers [133], to be an effective technique in increasing surface wettability of PTFE. The DBD plasma treated PTEF showed good adhesion and growth of endothelial cells (EC) that originated from the bovine pulmonary artery due to adhesion of fibronectin and vitronectin from the supplement serum of the cell culture medium. Furthermore, the spreading of ECs was better on DBD plasma treated PTEF than non-treated according to cell population densities. Table 3 represents an overview of different studies regarding endothelial cell cultivation on atmospheric pressure treated polymers.

### 4.4. Other Cell Lines 

Many researchers have studied cell cultivation of other than the mentioned above types of cells, including cardiac cells, epithelial cells, mesenchymal stem cells (MSC), on surface-modified polymers for biomedical applications. For instance, Lee and coworkers [135] used atmospheric radio-frequency plasma of different reactive gases (H_2_, O_2_, N_2_) with Ar as plasma gas to modify the PCL surface. Human prostate epithelial cells (HPEC) were cultured on the three surfaces, that were treated with different plasma gases (Ar/H_2_, Ar/O_2_, and Ar/N_2_). In vitro cell attachment and proliferation showed that Ar/H_2_ plasma-treated PCL substrate lowered initial cell loading as well as decreased the level of cell attachment and proliferation compared with the pristine substrate. That is due to Ar/H_2_ plasma-treated PCL substrate increased hydrophobicity, whereas Ar/N_2_ and Ar/O_2_ plasma-treated substrate promoted cell adhesion and showed better cell proliferation and growth.

Spence et al. [81] investigated surface modification of polyvinylidene fluoride (PVDF), ethylene-chlorotrifluoroethylene (ECTFE), and polyether ether ketone (PEEK) using the atmospheric pressure plasma treatment. MSC was cultured on PVDF, ECTFE, and PEEK and biological assays were performed. The cellular morphology, viability, differentiation, and cytoskeletal structure; showed that the viability, cellular activity and the attachment of spherically shaped MSC on atmospheric pressure plasma treatment treated surfaces, have enhanced.Moreover, Alem et al. [136] investigated MSC attachment and proliferation on PCL/Chitosan and PCL/carboxymethyl chitosan (CMC) scaffolds modified with helium cold atmospheric pressure (CAP). The results indicate that the helium CAP treatment improved cell attachment, and the scaffold could induce chondrocyte cell formation. Furthermore, PCL/CMC scaffolds were superior to PCL/Chitosan for cartilage tissue engineering applications because it supports cellular connectivity and cell proliferation.

Fabbri and coworkers [137] studied cell culturing of embryonic rat cardiac H9c2 cells on DBD air plasma modified substrate. Poly (butylene succinate)-based (PBS) copolymer containing thioether linkage (p(BS85BTDG15)) treated substrate biocompatibility assays showed the absence of cytotoxic products and proved that the treated surface can support cell adhesion and proliferation. Moreover, some investigations used more than one cell lines to study the effect of atmospheric pressure plasma treatment of polymeric materials. For instance, Revnickova et al. [138] investigated the enhancement of vascular smooth muscle cells (VSMCs) and mouse fibroblasts (L929) adherence on different polyethylene polymers. The low-density polyethylene (LDPE), high-density polyethylene (HDPE), and ultra-high molecular weight polyethylene (UHMWPE) were activated by Ar plasma discharges, and cytocompatibility of plasma treated polyethylene was studied. It was found that the cell number, morphology, and metabolic activity of the adhered and proliferated VSMCs and L929 cells were enhanced in plasma activated polyethylene foils. 

The following table, which is Table 4 represents the previously mentioned studies of different types of cell lines used in cell cultivation, characterization of different atmospheric pressure plasma surface modification techniques effectiveness. Moreover, it shows studies of atmospheric pressure plasma treated polymers with multiple cell lines cultivation characterizations.

## 5. Challenges and Future Perspective

The atmospheric pressure plasma treatment for polymer surface modification has gained a remarkable attention, owing to its advantages in enhancement of surface properties without affecting bulk properties. However, atmospheric pressure plasma treatment still faces barriers including non-uniformity, instability, inhomogeneity, and transmission into hot plasma during long treatment period. Furthermore, industrial scale processing of polymeric materials with atmospheric pressure plasma treatment has some limits, such as fast and uniform processing of large area. 

Furthermore, plasma parameters and plasma species that are necessary to functionalize polymer surface for good cell cultivation needed further works. The utilization of experimental diagnostics and modeling tools in order to better characterize atmospheric pressure plasma as a function of different operating parameters, such as plasma gas composition, is needed. Moreover, the control and generation of suitable active species for different cell line cultivation needed further experimental work. 

Developing a new surface modification technique based on different plasma techniques, with different grafting procedures is still a challenge, especially for polymer materials. Moreover, printing polymers to fabricate a 3D scaffold and incorporation specific chemical groups to print cells directly on the treated scaffold by atmospheric plasma treatment have a high potential in tissue engineering applications.

## 6. Conclusions

Tissue engineering is promising, challenging, and fast-developing interdisciplinary research field. Biomaterial science, especially surface modification, has been the interest of many research groups because it leads to promising advances in the tissue engineering field. Polymers are the most widespread materials being used in medical applications, due to their diversity, manufacturability, and suitable bulk properties, conclusions of this review article can be drawn as follows:

1. Polymers poor surface properties limit their utilization in medical applications. Over the last decade, extensive effort has been made in polymer surface modification techniques. One of the promising surface modification techniques is atmospheric pressure plasma treatment.
2. Atmospheric plasma treatment has significant benefits in comparison with traditional surface modification techniques including wet chemistry technique. For instance, short treatment duration, cost-effectiveness due to avoiding the cost of vacuum equipment, and simplicity are advantages of the atmospheric pressure plasma surface modification technique. Furthermore, the high density of reactive species in this technique, which enhances the formation of functional groups on polymer surface, such as carbonyl group -C=O, carboxyl group -C-O-OH, and hydroxyl group -C-OH, enhance cell-polymer interaction.
3. Cell-polymer interaction depends on surface topography, wettability, and cleanliness besides chemical properties.
4. As indicated in the review, that atmospheric pressure plasma surface modification technique of polymers has a satisfactory effect on enhancing cell-polymer interaction. Different types of cell lines exhibit enhancement in cell viability, adhesion, and proliferation.

## Figures and Tables

**Table 1:** A representative overview of atmospheric pressure plasma treatment of polymers and influence on fibroblast cell cultivation.

Substrate	Plasma Type	Plasma Gas	Grafted or Deposited Layer	Cell Type	Observation	Reference
PCL	APPJ	Ar	PCL homopolymer PCL/PEG copolymer	L929	Enhancement in cell adhesion, proliferation and growth for deposited PCL/PEG scaffold	[94]
PCL/Chitosan/PCL	DBD	Ar/O_2_ Ar/N_2_	-	L929	Increased cell viability, attachment and proliferation for Ar/O_2_ treatment.	[95]
PCL/Chitosan/PCL	DBD Plasma jet	Ar/O_2_ Ar/N_2_ Dry air	-	MRC5	Enhancement of initial cell attachment and seven-day cell viability, proliferation, and growth for both treatments in comparison with the untreated scaffold.	[96]
LDPE	DBD	Ar /O_2_	-	NIH 3T3	Improvement in adhesion and cytocompatibility of cells.	[98]
PLLA	Linear corona discharge	N_2_	_-_	MEF	Higher field/body area ratio Reduced percentage of apoptotic nuclei elongated with dendritic morphology	[17]
PTFE and PO_x_ copolymer	DCSBD	Ar/air	-	3T3	Increased cells adhesion number	[102]
PS	APPJ	Air	-	L929	Increase in the number of the attached fibroblast Wider spreading phenotype Vinculin protein and PTK2 increased	[103]

**Table 2:** A representative overview of atmospheric pressure plasma treatment of polymers and influence on osteoblast and osteosarcoma cell cultivation.

Substrate	Plasma Type	Plasma Gas	Grafted or Deposited Layer	Cell Type	Observation	Reference
**PCL**	DBD	O_2_	-	7F2	improves the initial attachment, proliferation, and migration	[18]
**PCL**	APPJ	He/O_2_ He	-	Saos-2	Clusterization of cells Presence of actin stress fibers	[113]
**PCL**	APPJ	Ar	APTES precursor	Human osteoblast	Improvement in cell viability after HVP functionalization and increase in cell number	[114]
**PLLA**	APPJ	Air CO_2_ C_3_F_8_	-	MC3T3-E1	Samples treated in air or CO_2_ gas were significantly superior in number and growth of adhering cells comparing to C_3_F_8_ treated substrate	[117,118]
**PLA**	APPJ	Ar	-	MG-63	cell proliferation and ALP activity significantly enhanced	[115]
**PE**	APPJ (microplasma)	He/O_2_	-	7F2	The cells attached and survived on the treated substrate	[121]
**UHMWPE**	APPJ	He/O_2_	-	MG-63	No significant difference between treated and untreated substrate	[122]
**PS**	APPJ	He/O_2_	PDMS precursor	MG-63	Enhancement of cell adhesion	[123]
**PMMA/PS**	DBD	Air	-	hFOB	Enhancement of cellular response	[124]

**Table 3:** A representative overview of atmospheric pressure plasma treatment of polymers and influence on endothelial cell cultivation.

Substrate	Plasma Type	Plasma Gas	Grafted or Deposited Layer	Cell Type	Observation	Reference
PCL	RFGD	Air	-	HCAEC	Introduction of -COOH group, that enhance gelatin grafting, and therefore increase cell adhesion, proliferation and maintain the expression of characteristic markers.	[130]
PLLA	Self-sustained barrier discharge	Air	-	HUVEC	Enhanced biocompatibility	[131]
PU	APP	He	-	HCAEC	Enhancement of HCAEC growth and adhesion under laminar flow.	[132]
PTFE	DBD	Air	-	EC	Good adhesion and growth of ECs Better spreading of ECs than the untreated substrate	[133]
PU PU/PLGA	MW induced	Ar	PU	HUVEC	PU/PLGA plasma treated substrate significantly increased the attachment of HUVEC a slightly enhanced the proliferation of the cells	[134]

**Table 4:** A representative overview of atmospheric pressure plasma treatment of polymers and its influence on the cultivation of different cell lines.

Substrate	Plasma Type	Plasma Gas	Cell Type	Observation	Reference
PCL	RF-plasma	Ar/O_2_ Ar/N_2_ Ar/H_2_	HPEC	Ar/N_2_ and Ar/O_2_ plasma-treated substrate improved adhesion properties and showed better cell proliferation and growth	[135]
PVDF ECTFE PEEK	APP	Air	MSC	Increased viability, cellular activity, and attachment on atmospheric pressure plasma treated surfaces. Spherically shaped MSC	[81]
PCL/Chitosan PCL/CMC	CAP	He	MSC	Improved cell attachment Induce chondrocyte cell formation.	[136]
PBS	DBD	Air	H9c2	Absence of cytotoxic products Support cell adhesion and proliferation	[137]
LDPE HDPE UHMWPE	Glow discharge plasma	Ar	L929 VSMC	Adhesion and proliferation of L929 cells were enhanced on all the plasma-treated samples. High viability values of VSMC in plasma treated substrate.	[138]
Flat PLA Honey-comb PLA	DBD-APPJ system	N_2_/O_2_ pretreatment N_2_/NH_3_ plasma treatment	NIH-3T3 Neuro-2A	Improve cell attachment and proliferation under all surface conditions.	[139]
PET	DBD	Air	Saos-2 HUVEC	Positive influence on the growth of both cell types	[140]
